# Genome-wide candidate regions for selective sweeps revealed through massive parallel sequencing of DNA across ten turkey populations

**DOI:** 10.1186/s12863-014-0117-4

**Published:** 2014-11-25

**Authors:** Muhammad L Aslam, John WM Bastiaansen, Hendrik-Jan Megens, Richard PMA Crooijmans, Fozia Nasreen, Le Ann Blomberg, Curtis P Van Tassell, Tad S Sonstegard, Steven G Schroeder, Martien AM Groenen, Julie A Long

**Affiliations:** Animal Breeding and Genomics Centre, Wageningen University, 6708 WD Wageningen, The Netherlands; National Institute for Genomics and Advanced Biotechnology, National Agricultural Research Centre, Islamabad, 44000 Pakistan; Animal Biosciences and Biotechnology Laboratory, Animal and Natural Resources Institute, Beltsville Agricultural Research Center, United States Department of Agriculture, Beltsville, MD 20705 USA; Bovine Functional Genomics Laboratory, Animal and Natural Resources Institute, Beltsville Agricultural Research Center, United States Department of Agriculture, Beltsville, MD 20705 USA

## Abstract

**Background:**

The domestic turkey (*Meleagris gallopavo*) is an important agricultural species that is largely used as a meat-type bird. Characterizing genetic variation in populations of domesticated species and associating these variation patterns with the evolution, domestication, and selective breeding is critical for understanding the dynamics of genomic change in these species. Intense selective breeding and population bottlenecks are expected to leave signatures in the genome of domesticated species, such as unusually low nucleotide diversity or the presence of exceptionally extended haplotype homozygosity. These patterns of variation in selected populations are highly useful to not only understand the consequences of selective breeding and population dynamics, but also to provide insights into biological mechanisms that may affect physiological processes important to bring changes in phenotype of interest.

**Results:**

We observed 54 genomic regions in heritage and commercial turkey populations on 14 different chromosomes that showed statistically significant (P < 0.05) reduction in genomic variation indicating candidate selective sweeps. Areas with evidence of selective sweeps varied from 1.5 Mb to 13.8 Mb in length. Out of these 54 sweeps, 23 overlapped at least partially between two or more populations. Overlapping sweeps were found on 13 different chromosomes. The remaining 31 sweeps were population-specific and were observed on 12 different chromosomes, with 26 of these regions present only in commercial populations. Genes that are known to affect growth were enriched in the sweep regions.

**Conclusion:**

The turkey genome showed large sweep regions. The relatively high number of sweep regions in commercial turkey populations compared to heritage varieties and the enrichment of genes important to growth in these regions, suggest that these sweeps are the result of intense selection in these commercial lines, moving specific haplotypes towards fixation.

**Electronic supplementary material:**

The online version of this article (doi:10.1186/s12863-014-0117-4) contains supplementary material, which is available to authorized users.

## Background

Characterizing genetic variation in populations of domesticated species and associating these variation patterns with the evolution, domestication, and selective breeding is critical for understanding the dynamics of genomic change in these species. Intense selective breeding and population bottlenecks are expected to leave signatures in the genome of domesticated species, such as unusually low nucleotide diversity or the presence of exceptionally extended haplotype homozygosity [1-3]. Genome-wide characterization of many different breeds and populations for these selective sweeps, along with the functional knowledge of the region, can reveal which genes are linked to traits or diseases with a complex genetic basis [4]. These patterns of variation in selected populations are highly useful to not only understand the consequences of selective breeding and population dynamics, but also to provide insights into biological mechanisms that may affect physiological processes important to bring changes in phenotype of interest [5,6].

The turkey (*Meleagris gallopavo*) is an important agricultural species that is largely used as a meat-type bird. All domesticated turkeys descend from the wild turkeys indigenous to North and South America. There are seven subspecies of the wild form [7] distinguished by geographic range and plumage differences. They are: South Mexican (*M. g. gallopavo*), Rio Grande (*M. g. intermedia*), Merriam’s (*M. g. merriami*), Gould’s (*M. g. mexicana*), Eastern (*M. g. silverstris*), Moore’s (*M. g. oneusta*) and Florida (*M. g. osceola*). Three of the seven are believed to play an important role in domestication. It is generally accepted that domestication of turkey involved South Mexican turkey [8]. The earliest signs of turkey domestication dates to approximately 2000 years ago at Mayan sites in Southern Mexico such as Cobá [9]. Domestic turkey stocks were established by at least 180 AD within the Tehuacán valley [10]. A separate domestication event likely occurred in what is now the Southwest United States, where the first strong archaeological evidence for domestic stocks dates to similar time (ca. 200 BC–AD 500), although the wild progenitor has been long debated [11].

The modern domestic turkey has been recognized by the American Standard of Perfection since 1971 [12], and is registered as a single breed with eight varieties defined primarily by plumage color. Out of these eight heritage turkey varieties, five (Bronze, Narragansett, White Holland, Spanish Black and Blue Slate) were registered in 1874 [12], while the remaining three (Beltsville Small White, Bourbon Red, and Royal Palm) were registered in 1951, 1909, and 1971 respectively [12]. These domestic turkeys are presumed to be highly inbred [12], and to have undergone intensive selection for traits of economic importance such as body weight, meat quality and egg production [9,11].

Recent census data show that turkey is the second largest contributor in worldwide poultry meat production [13]. Global production of turkeys has experienced a massive expansion over the past 40 years. In 2008, turkeys represented 6.65% of the world poultry meat production [13]. Global turkey stocks nearly tripled from 178 million in 1970 to over 482 million in 2008 [13]. Astonishingly, in those four decades, average meat production per bird doubled from 6.7 to 12.7 Kg, showing the result of intensive selection in turkeys.

An important genomic indicator of a selective sweep involves local reduction in genetic variation within the selected gene(s) and in nearby single nucleotide polymorphism (SNP) variants [14]. Selection affects all the genomic variability in the genome, including SNPs, microsatellites and several types of structural variations (SVs). The SV category includes large insertions and deletions, inversions, duplications and balanced or unbalanced inter-chromosomal translocations. Next generation sequencing (NGS) is an efficient approach for a large-scale, genome-wide SNP discovery and genotyping of individuals [15,16]. Availability of a high quality reference genome sequence [17] and resequencing of individuals or groups with appropriate genome coverage are key prerequisites for whole-genome SNP discovery [15,16]. Genomic sequences of individuals are aligned to a reference genome to detect nucleotide variations, i.e., differences in genotype of individuals at specific positions of the genome [18,19].

Our search was aimed at finding genomic regions where selection or domestication has changed the frequency of favourable alleles towards fixation. Genomic regions where these changes are observed elucidate the effect from the selective pressure of domestication or selection that was applied to the domesticated turkey.

## Methods

### Populations

Ten turkey populations that included seven commercial lines and three heritage varieties were used for whole genome sequencing (WGS). The seven commercial lines, L1 through L7, were provided by two breeding companies. Commercial lines were selected for different objectives including higher adult body weight and rapid growth except L5 which is a female line that was selected for medium adult body weight, conformation and egg production. The heritage varieties were Beltsville Small White (BvSW), Royal Palm (RP) and Narragansett (Nset) [20-22]. In total, 29 individuals were selected for WGS, with three individuals per population except for RP, which was represented by two individuals.

### Genomic DNA Extraction, Library Preparation and Sequencing

Genomic DNA was extracted from whole blood with the QIAamp DNA blood Midi Kit (Qiagen, Valencia, CA); the procedure included a proteinase K digestion followed by column purification. Integrity of high molecular weight DNA following the extraction was confirmed by agarose gel analysis. Genomic DNA was sheared using the Covaris S2 to yield an average fragment size of 450 bp, as determined with the Agilent Bioanalyzer 2100 (Agilent, Santa Clara, CA).

Genomic libraries were prepared with the Paired-end Sequencing Sample Preparation Kit (Illumina, San Diego, CA) with 5 μg of genomic DNA according to the manufacturer’s instructions. All genomic DNA libraries were validated with the Agilent Bioanalyzer (model 2100). The automated cBot Cluster Generation System (Illumina) was used to generate clusters on the flow cell. Each individual was sequenced (paired-end; read length 120 bp) in a single lane of a flow cell using the Illumina GAIIx.

### Sequence mapping

Sequence reads of each turkey were filtered on base quality; reads were trimmed if three consecutive bases had an average Phred-like quality score of less than 13. Both paired-end sequences of a fragment were required to be at least 40 bp long after trimming to be retained for analyses. Retained reads were aligned against the turkey reference genome (UMD 2.01) using the MOSAIK aligner [23]. Mapping of reads from each individual to the reference genome sequence was performed with hash size (hs) of 15, maximum hash positions (mhp) of 100, an alignment candidate threshold (act) of 20, and a maximum mismatch percentage (mmp) of 5. Banded Smith-Waterman algorithm (bw = 41) was used to increase the speed of alignments. The algorithm implemented in MOSAIK calculates a mapping quality for each sequence that measures the probability that a sequence belongs to a specific target. The alignments were filtered for ambiguously mapped reads and sorted using MosaikSort. Finally, the file was converted to BAM format [16] using MosaikText. All BAM files have been uploaded to NCBI's Sequence Read Archive (SRA) database under the study accession number “SRP012021” [24].

### Heterozygosity

Genome wide nucleotide diversity across the whole genome was assessed for each individual of the different turkey populations. The pileup function of SamTools version 0.1.12a [15] was used to perform SNP genotype calling, after which the nucleotide diversity was estimated across the whole genome for each individual separately. Nucleotide diversity was estimated by calculating the number of heterozygous SNP as well as the number of homozygous non-reference genotypes within each 300 Kb window. Windows of 300 Kb were necessary to avoid large random fluctuations in heterozygosity that were observed in a preliminary analysis with smaller windows. The random fluctuations with smaller windows were due to the low SNP detection rate. For calling SNPs, coverage per base was limited to 5-10 fold to avoid analysing repetitive regions of the genome as the average sequence depth per animal, at bases covered by at least one read, ranged from 2.07 to 6.72 [24]. In addition genotypes were only called when the genotype quality was at least 20. Observed number of heterozygous SNPs per nucleotide position was then averaged for each population within the window size of 300 Kb.

### Estimation of threshold values for calling sweeps

Turkey chromosomes were divided into bins of 300 Kb, and these bins were used to estimate threshold values to determine significance levels of sweep regions in the genome. Patterns of heterozygosity among these bins were investigated separately for each turkey population. A sweep region was defined when heterozygosity was below the threshold for at least 5 consecutive bins. To obtain the genome wide significance threshold (P <0.05), heterozygosity values of the bins were randomly permuted across the genome. Subsequently the threshold that would lead to exactly one significant region of 5 consecutive bins was determined for each of 7000 replicates. The distribution of these 7000 thresholds was used to obtain the 5% genome wide threshold. With this 5% threshold heterozygosity value, each population had a 5% probability of finding 1 sweep region by chance. A threshold of five consecutive bins was selected because preliminary results showed large regions of homozygosity in the turkey genome, and also to obtain stable statistics for heterozygosity. Using these threshold values, each turkey population was investigated for regions of low heterozygosity indicative of the presence of a sweep. Subsequently, turkey populations were compared with each other for the overlap in putative sweep regions. Overlapping sweep regions were identified when a sweep was replicated in more than one population. The overlapping sweep regions were defined as the genomic region covered by the sweeps from at least two populations.

### Heat map plots

Heat maps for the individual turkey chromosomes and for the whole turkey genome, including all turkey autosomes, were plotted separately to visualize overlapping sweeps in different turkey populations using the “heatmap.plus” package in R [25]. The color scale was based on the square root of heterozygosity values, for visualization and distinction of sweep areas in the genomic regions.

### Functional annotation analysis

All genes lying within the overlapping sweep regions of turkey were used for functional annotation analysis. Functional annotation analysis was performed using DAVID (Database for Annotation, Visualization, and Integrated Discovery) with default parameters [26]. DAVID is a web-based bioinformatics application that systematically identifies enriched biology associated with large gene list(s) derived from high-throughput genomic experiments [26]. Correction for multiple comparison was done by the Benjamini-Hochberg method [27]. Annotation for turkey and chicken genes is very limited; therefore we used one to one orthologs of turkey to human to perform this functional annotation analysis.

### Ethical approval for the use of animals in this study

Although animals were used in this work, no direct experiments were performed on them. Blood sample collection was carried out by highly skilled and experienced personnel from the breeding companies. No approval from the ethics committee was necessary according to local legislation.

## Results

In order to identify candidate selective sweeps, threshold values were estimated for heterozygosity in each of the different turkey populations. These threshold heterozygosity values ranged from 1.0E-5 to 5.1E-5 (Table 1). The highest threshold value was obtained for the L3 commercial line while the lowest threshold value was obtained for BvSW.

**Table 1:** **Estimates of heterozygosity threshold (P ≤0.05) of analyzed turkey populations**

**ID**	**Threshold** ^**1**^
Commercial Line L1	0.000030
Commercial Line L2	0.000022
Commercial Line L3	0.000051
Commercial Line L4	0.000014
Commercial Line L5	0.000029
Commercial Line L6	0.000032
Commercial Line L7	0.000026
Beltsville Small White	0.000010
Narragansett	0.000049
Royal Palm	0.000023

^1^Estimates of threshold values (P ≤0.05) to declare a candidate selective sweep when heterozygosity was found below the threshold in 5 consecutive bins of 300 Kb for each population.

A whole genome view of the sweep regions in the different turkey populations is presented in 1. In total, we observed 54 genomic regions where heterozygosity was significantly reduced (P <0.05). These candidate selective sweeps were found on 14 different chromosomes across turkey populations (Additional file 1). Areas with evidence of candidate selective sweeps varied from 1.5 Mb to 11.1 Mb in length (Additional file 1). Out of these 54 sweep regions, 31 were population-specific (Additional file 1) and observed on 12 different turkey chromosomes, while 23 were overlapping sweep regions that were observed in multiple populations and distributed across 13 different chromosomes (Table 2 & Additional file 1).

**Figure 1:**
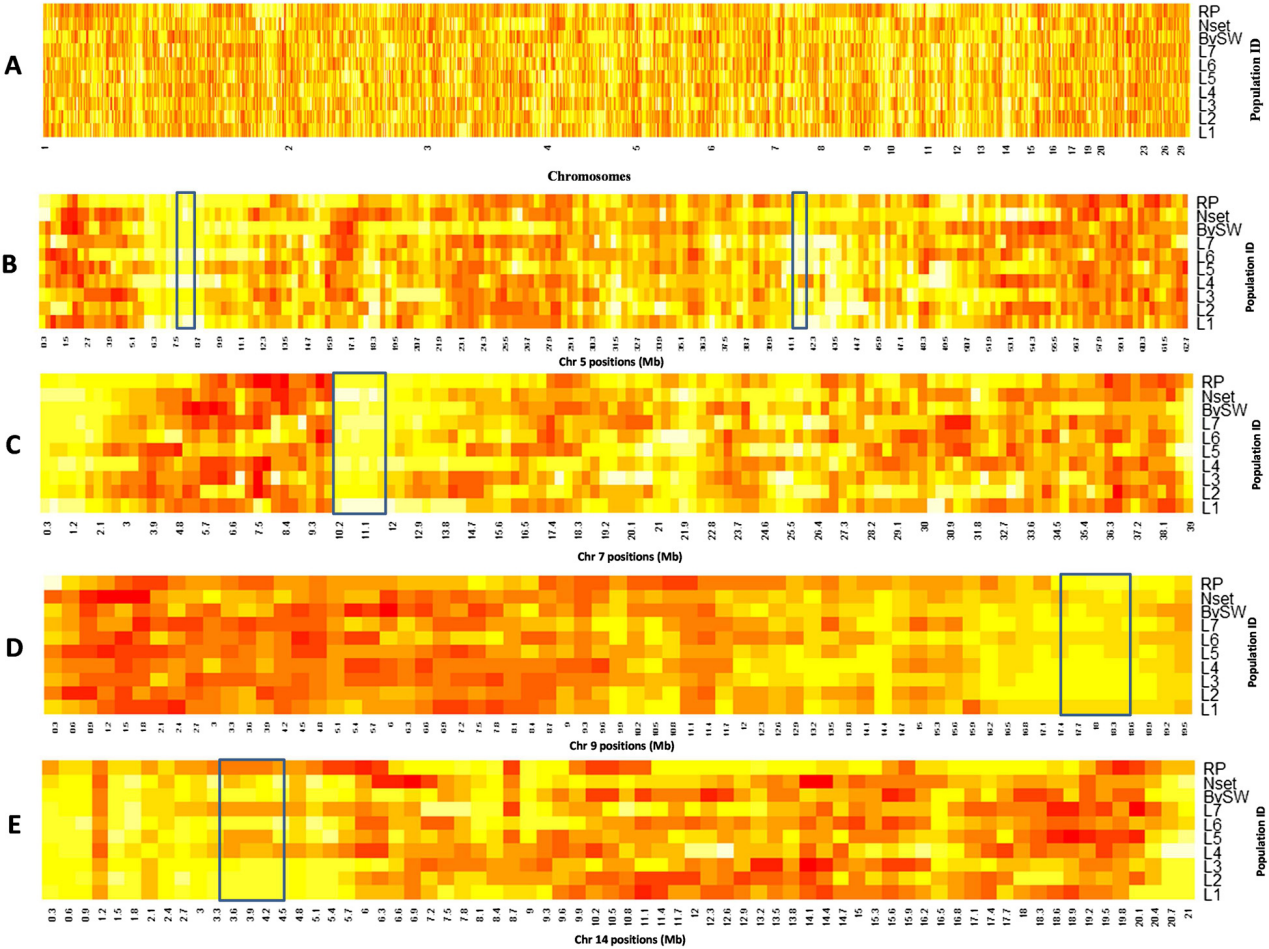
**Turkey chromosomes showing sweep regions shared by different turkey populations. A)** Turkey autosomes (1-30) showing variation in pattern of heterozygosity, colour pattern from white to red indicating a low to high level of heterozygosity. **B)** Turkey chromosome 5 with 2 candidate selective sweep regions from positions 7.8-8.4 Mb and 41.1-42.0 Mb shared by 5 different turkey populations (L1, L4, L6, BvSW, RP and L1, L3, L5, L6, L7 respectively). **C)** Turkey chromosome 7 with sweep region from positions 9.9-11.7 Mb shared by 4 different turkey populations (L1, L4, BvSW and RP). **D)** Turkey chromosome 9 with candidate selective sweep region from positions 17.4-18.6 Mb shared by 5 different turkey populations (L3, L5, L6, Nset and BvSW). **E)** Turkey chromosome 14 with sweep region from positions 3.3-4.5 Mb shared by 4 different turkey populations (L1, L3, L6 and BvSW).

**Table 2:** **Turkey candidate selective sweeps showing overlap in multiple turkey populations**

**Chr**	**Sweep region** ^**1**^	**Overlapping region** ^**2**^	**Populations**
1	179100000-181800000	180000000-181800000	Nset, RP
2	48600000-51300000	49500000-51300000	L5, L6
2	58200000-60300000	59700000-60300000	BvSw, L3
2	83700000-85200000	83700000-85200000	L1, L7
3	27600000-34200000	28800000-30300000	L1, L5
3	35100000-37200000	35700000-37200000	L1, L2, L4
3	96000000-97800000	96900000-97800000	L1, L2, L5
3	96900000-99900000	98400000-99600000	L2, L5, Nset
4	48600000-51000000	49200000-51000000	L2, L6
5	6900000-9000000	7800000-8400000	L1, L4, L6, RP, BvSW
5	41100000-42900000	41100000-42000000	L1, L3, L5, L6, L7
6	7800000-9900000	7800000-9600000	L1, L7
6	25200000-27900000	26700000-27900000	L2, L3
7	9900000-12600000	9900000-11700000	L1, L4, RP, BvSW
8	300000-3300000	1200000-3000000	L3, L5
9	12600000-14400000	13800000-14400000	L4, L6
9	15900000-19500000	15600000-16200000	L3, L5, L6
9	15900000-19500000	17400000-18600000	L3, L5, L6, Nset, BvSW
10	16800000-20100000	17400000-19200000	L2, L5, RP
11	1200000-8400000	4200000-7500000	L2, L4, L7, RP
11	7800000-12000000	9900000-12000000	L3, L4
14	3000000-4500000	3300000-4500000	L1, L3, L6, BvSW
22	300000-2100000	600000-2100000	L1, L3, L6

^1^Start and end positions of sweep regions at different turkey chromosomes. ^2^Start and end positions of the region where the sweep overlap from the populations mentioned in same row of last column.

The majority of the population-specific regions, 26 in total, were observed in the commercial populations (L1-L7), on average nearly 4 per population; whereas heritage populations (BvSW, Nset and RP) showed 1.6 population-specific sweeps per population. Differences between commercial populations were considerable, with as many as 8 sweep regions observed in population L3 and only one population-specific sweep region observed in population L6. Five population-specific sweep regions were observed in heritage varieties with 1 (RP) or 2 (BvSW and Nset) sweeps per population.

Out of 23 sweep regions that showed overlap in multiple populations, one was observed only in the heritage varieties (Nset and RP) while 13 were observed only in the commercial lines (Table 2). Commercial line L1 had the largest sweep region, 11.1 Mb, (Additional file 1) as well as the highest number (10) of overlapping sweep regions. The lowest number (3) of overlapping sweep regions was observed in the heritage variety Nset (Table 2).

Differences were observed along the turkey genome, regarding the presence of sweeps at different chromosomes. Out of 54 observed sweep regions at different turkey chromosomes, chromosome 2 showed the highest number of significant regions, 8 in total, while chromosome 14 showed the lowest number, 2 in total. Chromosomes 5, 7, 9 and 14 had five candidate selective sweep regions that showed an overlap in at least 4 different turkey populations (Table 2; Figure 1). Chromosome 5 had two overlapping sweep regions that were each shared by at least five populations, and one of these two regions was presented by commercial lines only (Table 2). Chromosome 9 also had a sweep region that was shared by five populations (Table 2 and Figure 1).

Overlapping sweep regions covered 5,452 genes, 34.7% of the total number of genes that were identified in turkey genome sequence [17]. BioMart website version 0.7 (http://www.biomart.org) was used to identify human orthologs for turkey genes. Out of these turkey genes, 3,858 were one to one orthologs with human genes and 3,832 turkey genes had a corresponding HUGO Gene Nomenclature Committee (HGNC) symbol in human genebuild (GRC37.p7). Finally, 3,718 of these genes with HGNC symbol had annotation information available in DAVID and were used in the functional annotation analysis. Functional annotation analyses resulted in 514 gene ontology (GO) terms with an Expression Analysis Systematic Explorer (EASE) P-value [28] of less than 0.1 (Additional file 2) which is a rather liberal threshold because it does not correct for multiple testing. The EASE P-value is a modified Fisher Exact P-value. GO terms that passed the significant threshold of 0.05 after Benjamini Hochberg (B-H) correction [27] are shown in Table 3. The most enriched (B-H corrected P <0.0005) was embryonic morphogenesis, while the other terms in Table 3 are related to nucleic acid binding. The nominally significant GO terms (P <0.10, Additional file 2) included a few more terms related with morphogenesis or growth but were not significant after B-H correction.

**Table 3:** **Gene ontology (GO) terms that passed significant threshold of 0.05 after Benjamini Hochberg correction**

**GO term**	**Annotation term**	**Benjamini Hochberg P-value**
GO:0048598	Embryonic morphogenesis	0.0005
GO:0001882	Nucleoside binding	0.0022
GO:0017076	Purine nucleotide binding	0.0029
GO:0001883	Purine nucleoside binding	0.0043
GO:0030554	Adenyl nucleotide binding	0.0045
GO:0000166	Nucleotide binding	0.0080
GO:0032553	Ribonucleotide binding	0.0091
GO:0032555	Purine ribonucleotide binding	0.0091
GO:0032559	Adenyl ribonucleotide binding	0.0155
GO:0005524	ATP binding	0.0168

## Discussion

We aimed at finding genomic regions with reduced heterozygosity, either resulting from strong selection in favor of specific alleles or other reasons such as genetic drift. For the discovery of these regions in different turkey populations (commercial lines and the heritage varieties), we used a modified whole genome heterozygosity distribution approach [2]. In a particular population, the occurrence of heterozygosity values equal or less than the threshold value (Table 1) within at least 5 consecutive bins (each with 300 Kb size) indicates a significant reduction in heterozygosity in that region. Use of large window size might have limited our access to highlight smaller significant sweep regions. This large window size was chosen due to the detection of a large number of consecutive sweep deserts in our preliminary analyses which might be due to species specific low heterozygosity and/or overall low sequence depth [24]. In general, heterozygosity in turkey is low with an estimated average heterozygosity of 1.07 SNPs Kb^-1^ [24], much lower than the observed heterozygosity in chicken, with 4.28 and 2.24 SNPs Kb^-1^ reported in two different studies [2,29]. We estimated threshold values separately for each turkey population. The threshold values (Table 1) can also be regarded as a measure of the level of genetic diversity in a particular population. In our study, we found the highest threshold value for commercial population L3, which is concordant with the highest observed genetic diversity and the highest number of SNPs discovered in this population in our previous study [24]. Similarly, the lowest threshold value was observed for BvSW, also concordant with the previously observed lowest genetic diversity and the lowest number of SNPs discovered in this population [24].

In our study, 48 significant regions (population-specific and overlapping) were observed in the commercial populations, while only 6 significant regions (population-specific and overlapping) were observed in the heritage populations (Additional file 1 & Table 2). The small number of individuals (2-3) used per population could not reveal the complete variation of a particular population but each of these individuals still belonged to a specific population, therefore population specific terminology was used for the group of individuals that belong to a same population. The high number of candidate selective sweeps in commercial lines can be explained as a result of the high selection intensities applied to these populations [30]. A lower number of sweep regions in heritage varieties may be due to a number of reasons, such as the admixture of populations, relatively high effective populations size in heritage varieties, or relatively less intensive and less specific directional selection applied to these varieties in comparison to commercial turkeys. Specific information about population admixture or effective population size of heritage varieties is limited, but these varieties were likely pure lines given the anecdotal information from the turkey breeders.

In our previous study, among the heritage varieties, Nset showed the highest heterozygosity followed by RP and BvSW respectively [24]. A consistent pattern was observed with a lower number of sweep regions and a higher threshold heterozygosity value for Nset compared to BvSW and RP. These differences in number of sweeps and threshold heterozygosity values for the different populations may also be an indication of difference in level of admixture or effective population size. The heritage variety BvSW showed the lowest threshold heterozygosity value and also the highest number of sweeps of all heritage varieties, which is consistent with the severe bottleneck that this population went through in 2000 (Alexandra Scupham, Personal communication). Similarly, Nset population showed highest threshold heterozygosity value and the lowest number of sweeps of all domesticated turkey populations which could represent a higher level of admixture or comparatively larger effective population size for this population. However, no historical information is available to support this.

Regions of sweep with variable but large sizes (1.5-11.1 Mb) were observed. Reduction in genetic diversity/heterozygosity at different locations in the genome can persist for a long time, and indicate selection across a long genomic region [31]. The size of a sweep region may vary with history of domestication, the type of population (inbred or outbred), intensity of selection within a particular population, population dynamics such as bottlenecks and drift. SNP analyses of domestic dogs and cats show large stretches of alternating heterozygous and homozygous regions as a consequence of domestication and breed development [32,33]. In most outbred species, a selected region would display local SNP homozygosity, compared to abundant polymorphism elsewhere in the genome [34].

Uneven distribution of homozygous regions can be expected across the genome due to selection pressure through natural or artificial means [1-3,35]. Chromosome 5, 7, 9 and 14 are highly distinct with overlapping regions in at least four different turkey populations (Table 2). This suggests that genomic regions on these chromosomes contain gene(s) which affect the traits that are important for turkey production. Turkey populations that showed overlap in sweeps on these chromosomes may be selected for specific objectives that all populations had in common or, alternatively, may have been developed from parents that already were homozygous for these sweep regions. Two candidate selective sweep regions discovered on chromosome 5 and chromosome 22 show overlapping stretches only in commercial populations (Additional file 1). These regions may contain genes involved in commercially important traits. The regions, however, are too large to identify the individual genes that may have been selected.

We could not use museum samples (South Mexican turkeys) in our current data which were included in our previous study [24] due to their very low available sequence depth. Average sequence depth at bases covered by at least one read in museum samples ranged from 1.38 to 1.81 [24] which is less than half the depth (5 to 10 -fold) that was used as the criterion for calling SNPs in all individuals of the current study. However, even though coverage was low, in our previous study [24] we identified genomic regions at four chromosomes with increased homozygosity of non-reference alleles in the museum samples. The domesticated populations were found to be fixed for the reference alleles at those same loci [24]. These genomic regions with high non-reference allele homozygosity were aligned with the candidate selective sweep regions of current study to find any overlap. Besides the region at chromosome 3, the other regions at chromosomes 9, 14 and 22 showed overlap with the detected sweep regions (Additional file 1) of current study. These sweep regions of chromosome 9, 14 and 22 show overlap in 5, 4 and 3 populations respectively. This concordance of results supports our hypothesis that these candidate sweep regions are likely result of selection in commercial populations.

Chromosome studies have revealed that the karyotype is more conserved among avian species than in other taxa, such as mammals, with most avian species showing a diploid chromosome number between 76 and 80 (http://www.genomesize.com). This suggests that chromosomal evolution or large-scale rearrangements affecting chromosome number occur at a low rate in birds, and as a result many chromosomes have remained more or less intact during avian evolution [36]. Comparative cytogenetic and linkage maps between turkey and chicken showed conserved synteny and close ancestral relationships [37,38] that support the hypothetical ancestral Galliform karyotype [39]. The strong structural and functional conservation between the turkey and the chicken genomes [40,41], as well as the similarities in breeding objectives, suggest that overlap in selective sweep regions between the two species could be expected. To test whether selective sweep regions are conserved between chicken and turkey, the orthology to chicken for all significant overlapping sweep regions of turkey was determined. These genomic regions were then examined for the presence of sweeps, based on two different studies in the chicken [2,42]. Selective sweep studies reported about 400 sweep regions [2,42] which is about 0.38 sweep per Mb in chicken genome. Thirteen out of the 23 overlapping candidate selective sweep regions identified in turkey also harbored a selective sweep reported in chicken. Rubin et al. [2] reported 40 highly significant chicken sweep regions with very low Z transformed heterozygosity (ZHp < -6). Two of these highly significant chicken sweeps mapped within the syntenic regions of turkey sweeps on chromosomes 7 and 11 (Additional file 1). Overall, the concordance of chicken sweep regions with turkey sweep regions was low. Approximately 0.32 chicken sweeps were observed per Mb within the total overlapping sweep length of turkey. This result shows no enrichment of chicken sweeps within the overlapping sweep regions of turkey.

Selective sweep regions are expected to have been involved in producing phenotypic variation for the traits of interest and intensive selection leads these regions towards fixation. To investigate the variation explained by these regions, we looked for available turkey quantitative trait loci (QTL) information within these regions [41]. We did not find overlap between the QTL regions from our previous study [41] and the candidate sweep regions in the current analyses. This discordance could be explained if QTL regions were still too much variable to be identified in a search for selective sweeps. Due to the limited availability of information on turkey QTLs and the presence of structural and functional conservation between the turkey and chicken genomes [24,38,40], overlapping regions of candidate selective sweeps (Table 2) of turkey were aligned with chicken genome sequence (WASHUC2) to determine their positions in the chicken genome (Additional file 3). The orthologous chicken regions were subsequently examined for the presence of reported chicken QTL for growth [43]. Many QTL were found to be overlapped with these genomic regions (Additional file 3). The frequency of chicken growth QTL for which the confidence interval overlapped with the turkey sweep regions was found to be 11.33 growth QTL per Mb of sweep region. This high frequency of chicken growth QTL overlapping with the turkey candidate selective sweep regions was however a result of the high number of growth QTL discovered in chicken. The sweep regions did not show an enrichment of chicken QTL compared to other parts of the genome.

Production censuses of turkeys from the last four decades show that turkeys have doubled in size. We had therefore expected to see a sweep in the region of the somatomedin, insulin-like growth factor 1 (IGF-1), which is well known to play an important role in muscle growth and development in various domesticated species [44-46]. However, we did not find a candidate sweep near the IGF-1 region on turkey chromosome 1 (56348061 bp-56402610 bp). This observation suggests that the sequence variation at the IGF-1 locus itself is not involved in regulating the level in turkeys. Previously, two QTL were detected for IGF-1 level in blood plasma in chicken at chromosome 1 and 2 [47,48]. These two chicken QTL regions, both are syntenic with turkey overlapping candidate sweep regions at chromosome 1 and 6, respectively (Additional file 3), showing that some genes are present within the candidate sweep regions that appear to affect the level of IGF-1 hormone in blood, which has been shown to regulate growth, reproduction, energy balance, cell proliferation and cell death [49].

Given the large increase in production per bird from 6.7 to 12.7 Kg in a 40 years period [13], intensive selection for growth must have taken place in turkeys. The likely candidate genes such as *IGF2, Pit1, AFABP, PRKAG3, GDF8* etc. that have been previously reported to affect growth were not present within the candidate sweep regions. Gene ontology (GO) enrichment analysis was therefore performed to see if the complete set of genes within the candidate sweep regions has been enriched for association with growth. We performed gene functional annotation analysis using DAVID. Gene-based enrichment analysis showed some enrichment of genes for regulation of development and morphogenesis within the candidate sweep regions (Additional file 2). We found significantly (Benjamini P <0.05) enriched GO term with embryonic morphogenesis (Table 3) and other suggestive terms (1 < P >0.05) with embryonic organ morphogenesis, body development, maintenance of growth etc. (Additional file 2). This shows that the observed candidate selective sweep regions of turkey are enriched with genes that are important for some factors in growth and development.

## Conclusion

The turkey genome showed large candidate sweep regions. The relatively high number of candidate selective sweep regions in commercial turkey populations compared to heritage varieties provided evidence of intense selection in these commercial lines. In addition, the enrichment of these candidate sweep regions with genes of importance to growth indicates that these regions may have been targets of selection for growth in these commercial lines, moving specific haplotypes towards fixation.

### Availability of supporting data

BAM files of all the individuals used in current study are available online at NCBI's Sequence Read Archive (SRA) database under the study accession number “SRP012021” [http://www.ncbi.nlm.nih.gov/Traces/sra/?study=SRP012021].

## Additional files

Additional file 1:
**Position of turkey genomic regions with candidate selective sweeps in different turkey populations.** Description: This file contains the start and the end positions of turkey sweep regions on different chromosomes for each of the populations.This file also shows the syntenic selective sweep regions in chicken that coincide with turkey overlapping sweeps. Additional file 2:
**Gene ontology (GO) terms observed with functional annotation analysis performed using the online tool DAVID.** Description: This file contains all GO terms included in the functional annotation analysis with their biological functions and enrichment P-values. This file also contains the names of genes in the sweep regions that are annotated with each of the GO terms. Additional file 3:
**Syntenic positions of turkey overlapping sweep regions with chicken.** Description: This file contains the start and the end positions of turkey overlapping sweep regions and their syntenic positions in chicken genome. This file also contains chicken growth QTL for which the confidence interval overlapped with the turkey sweep regions.
